# Prognostic model for atrial fibrillation after cardiac surgery: a UK cohort study

**DOI:** 10.1007/s00392-022-02068-1

**Published:** 2022-08-05

**Authors:** Sheng-Chia Chung, Benjamin O’Brien, Gregory Y. H. Lip, Kara G. Fields, Jochen D. Muehlschlegel, Anshul Thakur, David Clifton, Gary S. Collins, Peter Watkinson, Rui Providencia

**Affiliations:** 1 Institute of Health Informatics Research, University College London, London, UK; 2 St. Bartholomew‘s Hospital, Barts Health NHS Trust, West Smithfield, London, UK; 3 Department of Cardiac Anesthesiology and Intensive Care Medicine, German Heart Center, Berlin, Germany; 4 Department of Cardiac Anesthesiology and Intensive Care Medicine, Charité Universitätsmedizin Berlin, Berlin, Germany; 5 Outcomes Research Consortium, Cleveland, OH USA; 6 Liverpool Centre for Cardiovascular Science, University of Liverpool and Liverpool Heart and Chest Hospital, Liverpool, UK; 7 Department of Clinical Medicine, Aalborg University, Aalborg, Denmark; 8 Department of Anesthesiology, Perioperative and Pain Medicine, Brigham and Women’s Hospital, Harvard Medical School, Boston, MA USA; 9 Department of Engineering Science, University of Oxford, Oxford, UK; 10 Centre for Statistics in Medicine, Botnar Research Centre, NDORMS, University of Oxford, Oxford, UK; 11 NIHR Oxford Biomedical Research Centre, Oxford University Hospitals NHS Foundation Trust, Oxford, UK; 12 Nuffield Department of Clinical Neurosciences, John Radcliffe Hospital, University of Oxford, Oxford, UK

**Keywords:** Atrial fibrillation after cardiac surgery, Atrial fibrillation, Cardiac surgery, Electronic health records, Epidemiology, Risk prediction, United Kingdom, Prognostic model, Risk score

## Abstract

**Objective:**

To develop a validated clinical prognostic model to determine the risk of atrial fibrillation after cardiac surgery as part of the PARADISE project (NIHR131227).

**Methods:**

Prospective cohort study with linked electronic health records from a cohort of 5.6 million people in the United Kingdom Clinical Practice Research Datalink from 1998 to 2016. For model development, we considered a priori candidate predictors including demographics, medical history, medications, and clinical biomarkers. We evaluated associations between covariates and the AF incidence at the end of follow-up using logistic regression with the least absolute shrinkage and selection operator. The model was validated internally with the bootstrap method; subsequent performance was examined by discrimination quantified with the c-statistic and calibration assessed by calibration plots. The study follows TRIPOD guidelines.

**Results:**

Between 1998 and 2016, 33,464 patients received cardiac surgery among the 5,601,803 eligible individuals. The final model included 13-predictors at baseline: age, year of index surgery, elevated CHA_2_DS_2_-VASc score, congestive heart failure, hypertension, acute coronary syndromes, mitral valve disease, ventricular tachycardia, valve surgery, receiving two combined procedures (e.g., valve replacement + coronary artery bypass grafting), or three combined procedures in the index procedure, statin use, and ethnicity other than white or black (statins and ethnicity were protective). This model had an optimism-corrected C-statistic of 0.68 both for the derivation and validation cohort. Calibration was good.

**Conclusions:**

We developed a model to identify a group of individuals at high risk of AF and adverse outcomes who could benefit from long-term arrhythmia monitoring, risk factor management, rhythm control and/or thromboprophylaxis.

**Graphical abstract:**

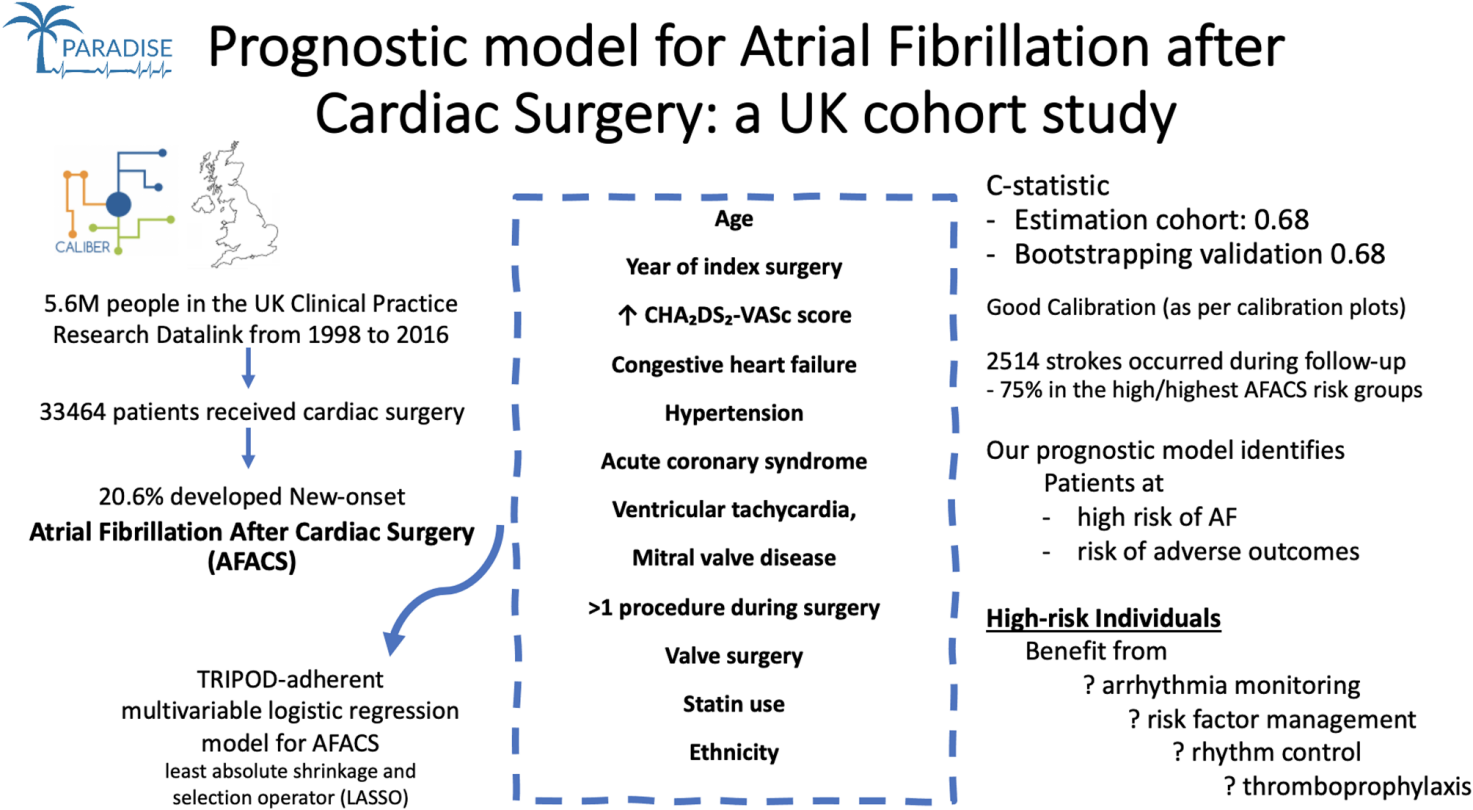

**Supplementary Information:**

The online version contains supplementary material available at 10.1007/s00392-022-02068-1.

## Introduction

Atrial fibrillation after cardiac surgery (AFACS) is frequent, occurring in 30–50% of cases [1]. Development of AFACS is associated with a number of perioperative pathophysiological and clinical factors [2]. Besides leading to longer hospital stays, AFACS has a prognostic impact, and has been associated with a higher risk of stroke, thromboembolism, and heart failure [3–5].

Interventions for preventing AFACS have shown to decrease AF incidence, length of hospital stay, and stroke [6]. Unfortunately, these treatments are not devoid of side effects. The *Society of Cardiovascular Anesthesiologists* and the *European Association of Cardiothoracic Anaesthetists* recommend different perioperative management depending on AFACS risk [7, 8]. However, there is no effective or validated way of risk stratifying these patients.

Strategies to identify patients at risk of developing AFACS would therefore be of interest and would allow the implementation of preventive strategies and more intensive monitoring to high-risk individuals. Some attempts have been made to develop prognostic schemes, but the currently available models [9–11] do not meet TRIPOD quality criteria [12].

A validated multivariable prognostic model allowing targeted prophylaxis is the first step to improving outcomes, informing patients, and resource planning. The objective of the study is to develop and validate a clinical prognostic model to determine the risk of a patient developing AFACS at the time of cardiac surgery.

## Methods

This investigation is part of a project addressing a *National Institute of Health Research* (NIHR) call on AFACS—HTA no 19/132: *“Predicting AF after Cardiac Surgery*–*the PARADISE Score. A Clinical Prediction Rule for Post-operative Atrial Fibrillation in Patients Undergoing Cardiac Surgery (PARADISE)”*; NIHR131227. The PARADISE project aims to develop validated clinical prediction models to determine the risk of a patient developing AFACS: in the pre-operative assessment clinic or on admission for surgery (PARADISE-1) and on arrival in the post-operative care unit (PARADISE-2). In this paper, we will identify variables from a linked electronic health record dataset associated with AFCAS. In the near future, we will subsequently test these variables in British and American datasets and trials, with the aim of developing the PARADISE-1 score. Both PARADISE 1 and 2 will subsequently be validated in British, US-American and German real-world patient cohorts.

### Study design, source of data, and population

We used a prospective cohort study to assess AF incidence among individuals with cardiac surgery. The Clinical Practice Research Datalink (CPRD) was established in 1987 and as of 2018 includes 7,998,501 patients in the UK with linked data of primary care consultation, hospital data (Hospital Episodes Statistics), national cancer registry (National Cancer Intelligence Network) and death registry data (Office for National Statistics—ONS) [13]. The data are generally representative of the age, gender, and geographic distribution of the UK population [13]. Previous validation studies of the UK nationwide EHR showed high quality and completeness of clinical information recorded in the data [14, 15]. The data used for the present study were approved by the MHRA (UK) Independent Scientific Advisory Committee [17_205], under Section 251 of the National Health Service (NHS) Social Care Act 2006. CALIBER has research ethics approval (09/H0810/16) and ECC approval (ECC 2-06(b)/2009 CALIBER dataset). The study followed the Transparent Reporting of a multivariable prognostic model for Individual Prognosis or Diagnosis (TRIPOD) recommendations [12].

**Fig. 1:**
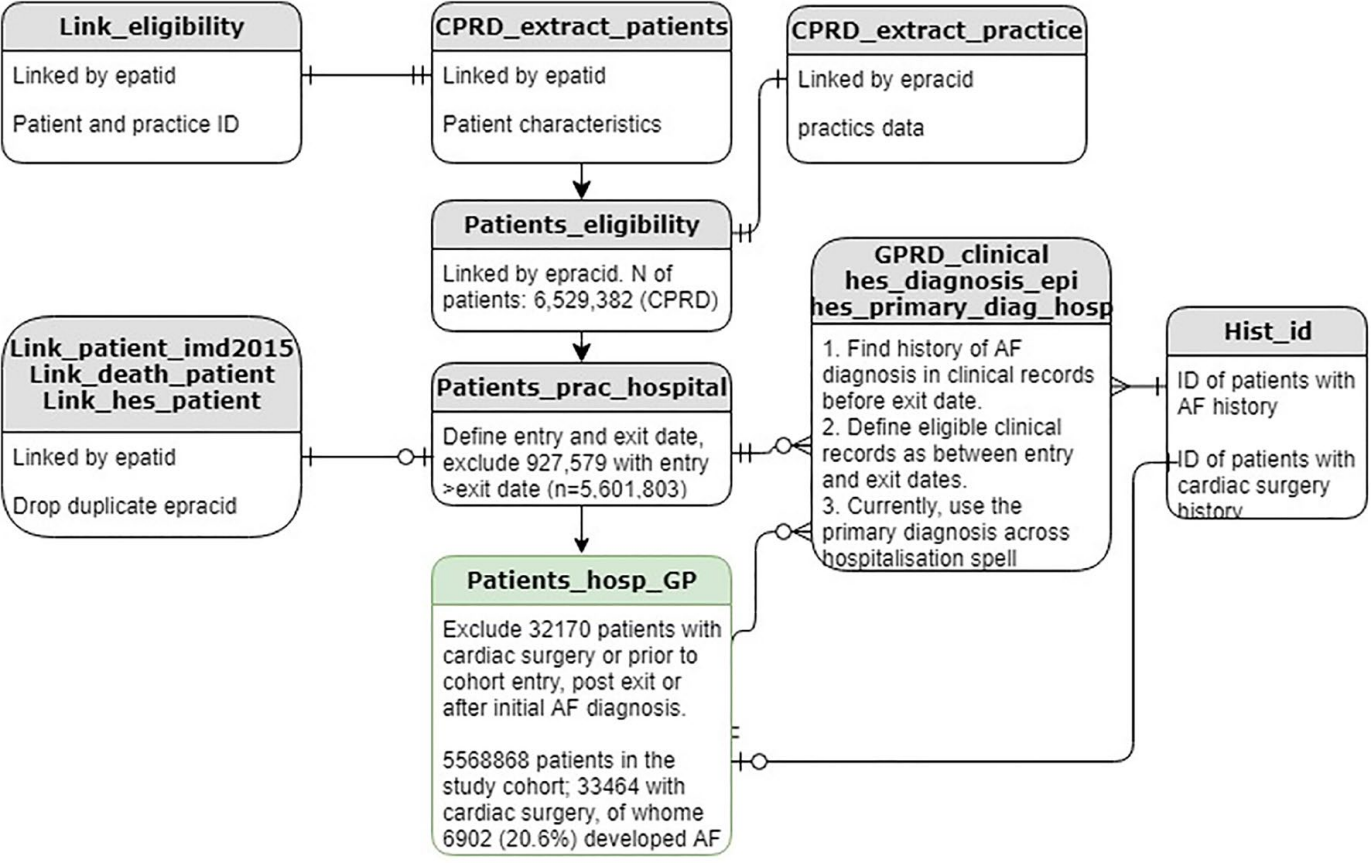
Study population

We identified individuals aged 18 years or older that had been registered in the current primary care practice for at least 1 year. The study period was between January 1, 1998, and May 31, 2016, and there were 401 practices included in the data (Fig. 1).

### Participants

Individuals who underwent cardiac surgery were included in the study. The definition of cardiac surgery was based on the Office of Population Censuses and Surveys Classification of Surgical Operations and Procedures (4th revision, OPCS-4) [16] in hospitalisation and their corresponding primary care READ coding. The complete definition was summarised in supplementary table S1. Individuals were excluded if they had a prior history of AF or cardiac surgery before study entry. Follow-up ceased for the following reasons: death, the end date of registration with the practice, last day of the general practice data collection or the end of the study period (31 May 2016).

### Outcome

The primary outcome was new-onset AF occurring after the date of cardiac surgery (AFACS). AF was defined from the International Classification of Diseases, tenth revision as I48 from HES and Read codes G573400, G573500, 3272.00, G573000, G573300, G573.00, G573z00 from CPRD. Based on the definition, AF cases included a minor proportion of atrial flutter [17]. The definition was developed and tested in cardiovascular disease research using linked bespoke studies and the electronic health records (CALIBER) platform (https://caliberresearch.org/portal) [18]. Previous research has shown high validity and completeness of the disease definition in AF and other conditions [19, 20]. We identified death, date of death and causes of death from the Office of National Statistics (ONS) records. New-onset AF included individuals with first AF diagnoses as the primary cause of death.

### Predictors

We used the Index of Multiple Deprivation (IMD) 2015 quintile to describe socioeconomic status, with a higher quintile representing the more deprived areas [21]. For eligible participants, we identified 45 predictors reported in the literature [22], reported in a Delphi process [23] or with high prevalence observed in the study cohort: age at cardiac surgery, sex, ethnicity categories, smoking status, history of diabetes, hypertension, stable angina, unstable angina, myocardial infarction, stroke, dementia, heart failure, chronic obstructive pulmonary disease, chronic kidney disease, cancer, asthma, valvular disease, deep vein thrombosis, pulmonary embolism, mitral valve disease, supraventricular tachycardia, cardiogenic shock, ventricular tachycardia, transient ischemic attack, hypothyroidism, dyslipidaemia, systolic blood pressure, diastolic blood pressure, calendar year at study entry [24], the use of anticoagulants, antiplatelet drugs, anti-arrhythmic drugs, beta-adrenoceptor blocking drugs, diuretics, warfarin, hypertension and heart failure drugs, statin, NSAIDS, cardiac glycosides, immunosuppressants, inotropic drugs. The CHA_2_DS_2_-VASc score was calculated [25], as a measure of stroke risk although prior studies have examined the role of this score in predicting AFCAS [26]. CHA_2_DS_2_-VASc Score and categorised into elevated CHA_2_DS_2_-VASc Score (≥ 1 for men and ≥ 2 for women). We reported the proportion of individuals with a diagnosis recorded in their primary care or hospital admissions, before their initial documentation of cardiac surgery. Diagnosis code lists for each condition were adapted from the CALIBER code repository (Supplementary table S2).

### Statistical analyses

Baseline characteristics were presented among derivation and validation groups. We reported frequencies (%) for categorical data and means with standard deviation for continuous data, and chi-square and *t* tests were used to examine the difference between sex and socioeconomic categories. The extent of missing values was assessed and then managed by recoding for categorical variables or multiple imputations for continuous variables.

### Model construction

Univariable models were used to examine non-linear trends. We used multivariable logistic regression with the least absolute shrinkage and selection operator (LASSO) for model building [27]. Variance inflation factors were calculated to detect evidence of multicollinearity problems in the model selection process. The minimum sample size for developing the was estimated [28]. Assuming a 0.21 prevalence of AF post cardiac surgery, 50 parameters (including dummy variables of predictors), and a Cox-Snell *R*^2^ of 0.0208. We estimated the minimum sample size required for new model development was 21,384.

### Model validation and performance

Model validation was implemented by internal bootstrap validation [29]. Model performance was examined by assessing discrimination and calibration in the study cohort. Discrimination was quantified with the c-statistic. Model calibration was assessed by calibration plots [30] and Hosmer–Lemeshow goodness-of-fit tests.

Additional analyses were performed using random forests for variable selection in constructing the prognostic model, with the 100 trees built from the randomly split training (75% of the full cohort) and validation (25% of the full cohort) populations. We performed the analyses in the secured Data Safe Haven, meeting the data safety and information governance requirements by University College London, NHS Digital and ONS. Analyses were performed in SAS (version 9.4), R (version 3.6.1) and Stata (version 16.1). The funders did not have any role in the study design, data collection, data analysis, interpretation, and writing of the report.

### Public and patient involvement

Patients were involved in the Delphi process for the selection of potential candidate variables, and will be involved in the dissemination of the study results.

## Results

The mean age of the 33,464 patients who received cardiac surgery was 57.3 years (standard deviation 13.5 years). 29.2% of the patients were women. Frequent comorbidities included hypertension (prevalence at baseline: 41.1%), ischaemic heart disease (38.7%), angina (37.3%), myocardial infarction (30.4%), and valvular disease (23.2%). (Table 1).

**Table 1:** Patient characteristics at study entry

	All (*n* = 33,464)	AF (*n* = 6902)	No AF (*n* = 26,562)	*p* value		All (*n* = 33,464)	AF (*n* = 6902)	No AF (*n* = 26,562)	*p* value
Age (years), mean, SD	55.7, 13.5	68.5, 10.9	62.5, 13.7	< 0.001	Medication, *n* (%)				
Women, *n* (%)	9775 (29.2%)	2045 (29.6%)	7730 (29.1%)	0.39	Anticoagulants	237 (0.7%)	38 (0.6%)	199 (0.7%)	0.08
Socioeconomic status quintile				0.11	Antiplatelet drugs	18,150 (54.2%)	3762 (54.5%)	14,388 (54.2%)	0.62
Quintile 1 (least deprived)	5507 (16.5%)	1201 (17.4%)	4306 (16.2%)		Antiarrhythmic drugs	1266 (3.8%)	283 (4.1%)	983 (3.7%)	0.12
Quintile 5 (most deprived)	7699 (23%)	1509 (21.9%)	6190 (23.3%)		Beta-blockers	16,076 (48%)	3271 (47.4%)	12,805 (48.2%)	0.23
Smoking	14,681 (43.9%)	1208 (47.2%)	11,875 (44.7%)	< 0.001	Diuretics	11,353 (33.9%)	2790 (40.4%)	8563 (32.2%)	< 0.001
Systolic blood pressure (mmHg), mean	135.9 (14.7)	138.2 (15.0)	135.3 (14.6)	0.14	Warfarin	1767 (5.3%)	330 (4.8%)	1437 (5.4%)	0.037
Diastolic blood pressure (mmHg), mean	77.6 (7.6)	77.2 (7.5)	77.7 (7.6)	< 0.001	Antihypertensive medication	14,851 (44.4%)	3171 (45.9%)	11,680 (44%)	0.003
CHA_2_DS_2_-VASc: ≥ 1 for men or ≥ 2 for women	6201 (89.8%)	2291 (89.6%)	21,091 (79.4%)	< 0.001	Statin	17,753 (43.1%)	3465 (50.2%)	14,288 (53.8%)	< 0.001
Comorbidities, *n* (%)					NSAID	17,063 (51%)	3577 (51.8%)	13,486 (50.8%)	0.12
Hypertension	13,752 (41.1%)	3233 (46.8%)	10,519 (39.6%)	< 0.001	Cardiac glycosides	925 (2.8%)	137 (2%)	788 (3%)	< 0.001
Diabetes	5820 (17.4%)	1155 (16.7%)	4665 (17.6%)	0.11	Immunosuppressants	247 (0.7%)	57 (0.8%)	190 (0.7%)	0.34
Valvular heart disease	7750 (23.2%)	2214 (32.1%)	5536 (20.8%)	< 0.001	Inotropes	831 (2.5%)	195 (2.8%)	636 (2.4%)	0.04
Angina	12,476 (37.3%)	2691 (39%)	9785 (36.8%)	0.001	Procedures, *n* (%)				
Ischemic heart disease	12,960 (38.7%)	2756 (39.9%)	10,204 (38.4%)	0.021	CABG	22,233 (66.4%)	4620 (66.9%)	17,613 (66.3%)	0.32
Heart failure	2815 (8.4%)	842 (12.2%)	1973 (7.4%)	< 0.001	Valve repair, replacement and valvotomy	8326 (24.9%)	2451 (35.5%)	5875 (22.1%)	< 0.001
Stroke	1534 (4.6%)	335 (4.9%)	1199 (4.5%)	0.23	Other cardiac surgery	21,940 (65.6%)	4381 (63.5%)	17,559 (66.1%)	< 0.001
Asthma	3319 (9.9%)	679 (9.8%)	2640 (9.9%)	0.8	Number of procedures				< 0.001
Hyperthyroidism	396 (1.2%)	88 (1.3%)	308 (1.2%)	0.43	1	16,254 (48.6%)	2969 (43.0%)	13,285 (50.0%)	
Cancer	3551 (10.6%)	870 (12.6%)	2681 (10.1%)	< 0.001	2	15,385 (46.0%)	3316 (48.0%)	12,069 (45.4%)	
Chronic kidney disease	2741 (8.2%)	594 (8.6%)	2147 (8.1%)	0.16	3	1825 (5.5%)	617 (8.9%)	1208 (4.6%)	
Chronic obstructive pulmonary disease	1546 (4.6%)	385 (5.6%)	1161 (4.4%)	< 0.001					
Dementia	107 (0.3%)	17 (0.2%)	90 (0.3%)	0.23					
Transient ischemic attack	1443 (4.3%)	339 (4.9%)	1104 (4.2%)	0.006					
Pulmonary embolism	539 (1.6%)	134 (1.9%)	405 (1.5%)	0.014					
Deep vein thrombosis	756 (2.3%)	192 (2.8%)	564 (2.1%)	0.001					
Peripheral artery disease	2536 (7.6%)	611 (8.9%)	1925 (7.2%)	< 0.001					
Myocardial infarction	10,161 (30.4%)	2153 (31.2%)	8008 (30.1%)	0.092					
Mitral valve disease	2567 (7.7%)	803 (11.6%)	1764 (6.6%)	< 0.001					
Supraventricular tachycardia	444 (1.3%)	104 (1.5%)	340 (1.3%)	0.14					
Ventricular tachycardia	743 (2.2%)	191 (2.8%)	552 (2.1%)	< 0.001					
Cardiogenic shock	12 (0%)	1 (0%)	11 (0%)	0.29					

*NSAID* non-steroid anti-inflammatory drugs, *CABG* coronary artery bypass grafting

There were 6902 (20.6%) individuals with AFACS reported. The incidence of AF was 20.8% in patients receiving bypass surgery and 29.4% among patients receiving valve surgery.

**Fig. 2:**
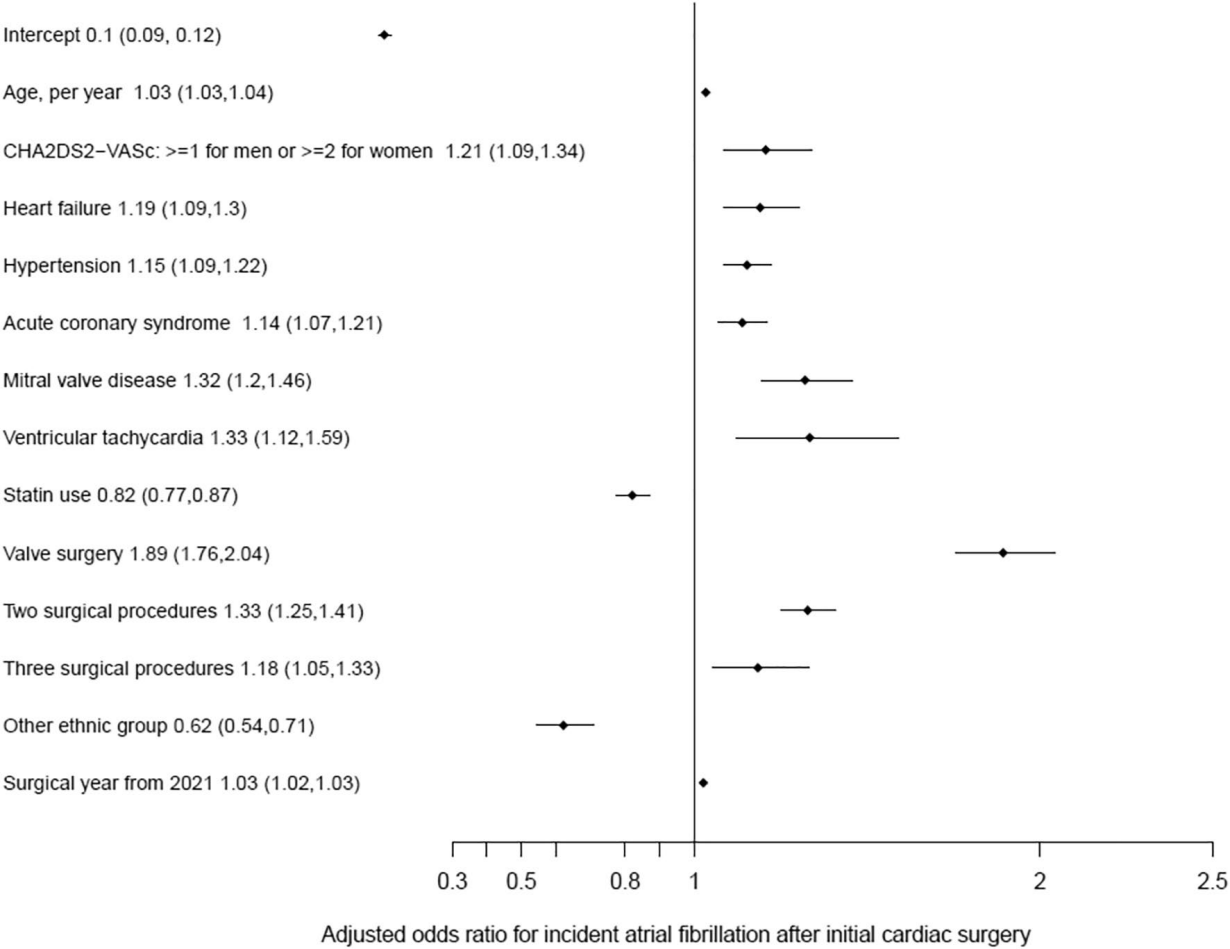
Summary of the adjusted odds ratio of prognostic factors

The C-statistic of the full model with 49 covariates was 0.69. LASSO selection of variables concluded in a final model with 13 predictors at baseline: including age (centred at 60 years), elevated CHA_2_DS_2_-VASc score, congestive heart failure, hypertension, acute coronary syndromes, mitral valve disease, ventricular tachycardia, statin use, valve surgery, receiving two combined procedures (e.g., heart valve repair + coronary artery bypass grafting), or three combined procedures (e.g., dual heart valve replacement + coronary artery bypass grafting) in the index cardiac surgery, ethnicity other than white or black, and year of the index surgery (Fig. 2). Statin use and ethnicity other than white or black were associated with a protective effect. Valve surgery as a type of cardiac surgical procedure was the strongest predictor of AFACS.

**Fig. 3:**
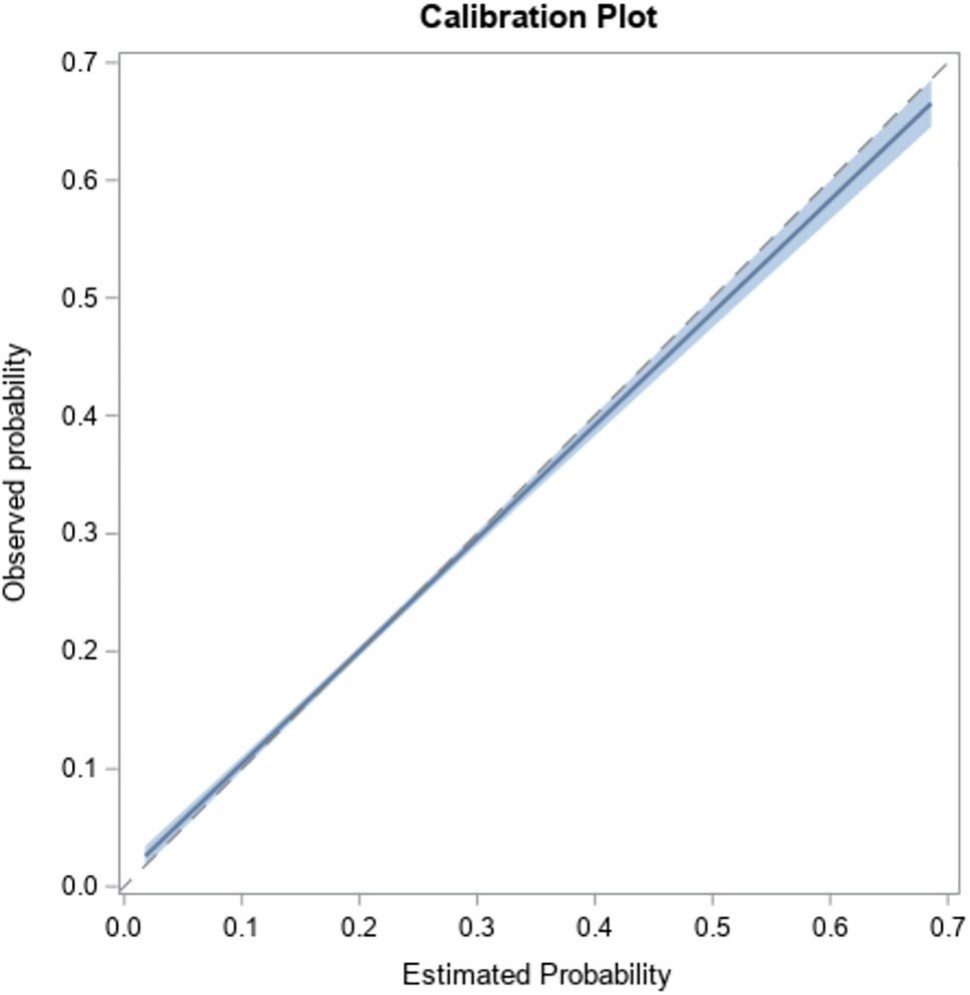
Calibration plots of the prognostic model

This model had a C-statistic of 0.68 in the estimation cohort. The C index for the prognostic model in the bootstrapping validation was 0.68, confirming the model has a moderate discrimination ability to identify patients at risk of AFACS. The model was well calibrated (Fig. 3). Sensitivity analyses with random forest model selection reported similar performance to the prognostic model (with the area under the curve: 0.64) (Supplementary Figs. 1, 2).

Analysis of the performance of the model showed that three quarters of the 7750 patients with valvular heart disease were classified as having a high or highest risk for developing new-onset AFACS (Table S3). Most cases (nearly 90%) of the 2214 new-onset AFACS events in patients with valvular heart disease were detected in the groups with high or highest AF risk as defined by our model (Table S4).

The AFACS risk profile of patients without valvular heart disease was lower, with more than half being assigned to the low or lowest risk groups. However, most of the new-onset AF cases in this group was also detected in the highest AFACS risk classes as defined by our model.

Finally, we estimated subsequent stroke occurrences in the different Predicted AFACS risk groups, as defined by our model, and observed that of the 2514 strokes occurring during follow-up ¾ were observed in the high or highest AFACS risk groups (Table S5).

## Discussion

In this multi-center cohort study, we successfully developed and validated an AF incidence prediction model for patients receiving cardiac surgery. The model showed adequate validity with a C-index of 0.68 and had good calibration for predicting AFACS.

From the predictors of AFACS in the model, we found that older age, elevated CHA_2_DS_2_-VASc score, history of congestive heart failure, hypertension, acute coronary syndromes, mitral valve disease, ventricular tachycardia at the time of cardiac surgery and earlier year of surgery performed, receiving valve surgery or multiple procedures performed during the index cardiac surgery were significantly associated with the subsequent AFACS, as documented in previous literature. We also found that statin use and ethnicity other than white or black were more likely to have a better prognosis (i.e., protective factors). These results have been consistent with previous reports.

Individuals with a higher risk of AFACS were more frequently diagnosed with AF and sustained more strokes during follow-up. This suggests that individuals with a higher risk of AFACS, as identified through our model, may represent a sub-population in whom mid to long-term arrhythmia monitoring (e.g., with implantable loop recorders) may be of interest, and measures like risk factor management, rhythm control and/or anticoagulation should be tested in future trials.

This study has several strengths. First, the large sample size of a total of 33,464 patients provides sufficient power in its results. Second, by including all patients receiving cardiac surgery the outcome can be representative of the national population of patients. Third, the use of a wide range of predictor variables, including socio-demographic data and data on clinical factors, and fourth the reproducibility of the model has been confirmed by bootstrapping validation and the performance was better than the modelling method using random forest. Our model also has several limitations. Detailed biomarker information, and information on clinical types of AF (paroxysmal or persistent), is not available for all patients. However, even though it is important to understand whether AFACS has a different prognosis or clinical impact depending on whether it is self-limited, behaving like paroxysmal AF (the most frequent occurrence according to Lin et al. [31]), or if it progresses to a more persistent form, the aim of our investigation was to develop a predictive model for AFACS, new-onset AF after cardiac surgery, irrespectively of its subsequent behaviour or burden. It is possible that the inclusion of additional variables could have improved the discrimination of the model and can be investigated in future research. The C-index of 0.68 is indicative of the multifactorial nature of risk for patients receiving cardiac surgery who subsequently developed AF. However, since strong statistically significant predictors were found, we can still draw important conclusions about how changes in the predictor values are associated with changes in the outcome, consistent with the literature.

### Implications and future research

Clinicians can utilize information from electronic health records to identify patients with a greater AFACS risk to aid the prioritisation of preventive care. This study will feed into the PARADISE-1 risk score, which aims at identifying patients at risk of AFACS prior to cardiac surgery. Combining some of the identified variables with biomarkers and imaging parameters may lead to better discrimination. The performance of any future scores can also be improved by adding intra-operatory variables or measures (as planned for PARADISE-2). Our research also supports future studies on other clinical outcomes, such as cerebrovascular ischemic events, following cardiac surgery, and interventions aimed at preventing them. An important aspect to clarify in future studies, as prevention treatment in the AFACS population starts to be considered, is the impact of AF burden or AF clinical type (paroxysmal vs. persistent) on subsequent clinical outcomes. This knowledge gap was highlighted in a systematic review by Lin et al. [31] assessing the impact of AFACS on mortality and stroke. The authors highlighted that none of the 35 included studies separately reported events for paroxysmal and persistent AF.

## Conclusion

We established and validated a prognostic model for new-onset atrial fibrillation among patients who received cardiac surgery. This model, or a future iteration utilizing some of its variables, can be useful to identify a group of high-risk individuals who could benefit from mid to long-term arrhythmia monitoring, risk factor management, rhythm control and/or thromboprophylaxis.

## Supplementary Information

Below is the link to the electronic supplementary material.

Supplementary file1 (DOCX 663 KB)
