# Supplementary material for: Prognostic model for atrial fibrillation after cardiac surgery: a UK cohort study
Source: Clin Res Cardiol. 2022 Aug 5;112(2):227–35. doi: 10.1007/s00392-022-02068-1 (PMC9898166; doi:10.1007/s00392-022-02068-1)
Supplement: Supplementary file 1 — Supplementary file1 (DOCX 663 KB) [file 392_2022_2068_MOESM1_ESM.docx]

**Supplementary Appendix**

Table of contents:

Table S1 definition of cardiac surgery

Table S2: Clinical codes used for defining AF and comorbidity in the study.

Table S3: Distribution of patients through the different predicted AFACS risk categories according to valvular heart disease status at baseline

Table S4: Distribution of confirmed AFACS cases by predicted risk groups in patients with baseline valvular heart disease

Table S5: Subsequent stroke occurrences in the different predicted AFACS risk groups

Figure S1: Importance of each predictor assessed by mean decrease accuracy and mean decrease Gini.

Figure S2: The ROC (Receiver Operating Characteristic) curve for the random forest classifier performance.

Supplementary table S1 definition of cardiac surgery

| Cardiac surgery | Hospitalisation (ICD10) | Primary care (CPRD Medcode) |
| --- | --- | --- |
| Coronary artery bypass graft | K40 K40.1 K40.2 K40.3 K40.4 K40.8 K40.9 K41 K41.1 K41.2 K41.3 K41.4 K41.8 K41.9 K42 K42.1 K42.2 K42.3 K42.4 K42.8 K42.9 K43 K43.1 K43.2 K43.3 K43.4 K43.8 K43.9 K44 K44.1 K44.8 K44.9 K45 K45.1 K45.2 K45.3 K45.4 K45.5 K45.8 K45.9 K46 K46.1 K46.2 K46.3 K46.4 K46.8 K46.9 | 737 3159 5744 7134 7137 7442 7609 7634 8312 8679 9414 10209 11610 12734 18249 19193 19402 19413 22647 28837 31519 31556 32651 33471 33718 34963 36011 37682 37719 42708 44561 45370 45886 48767 51507 51515 55092 55598 56990 57241 59423 60753 61310 62608 66236 66664 67591 67761 68123 68139 69776 70111 70755 72780 92419 93828 96804 |
| Valve repair or replacement | K25 K25.1 K25.2 K25.3 K25.4 K25.5 K25.8 K25.9 K26 K26.1 K26.2 K26.3 K26.4 K26.5 K26.8 K26.9 K27 K27.1 K27.2 K27.3 K27.4 K27.5 K27.6 K27.8 K27.9 K29 K29.1 K29.2 K29.3 K29.4 K29.5 K29.8 K29.9 K31 K31.1 K31.2 K31.3 K31.4 K31.5 K31.8 K31.9 K34 K34.1 K34.2 K34.3 K34.4 K34.8 K34.9 K36 K36.1 K36.8 K36.9 K38.3 | 1756 3169 3731 3911 4900 5643 7276 7894 9396 9498 15133 15910 16544 16636 17141 17812 19390 26153 26168 29887 30173 30567 30705 32930 35812 36638 36734 39763 40086 41168 43778 44690 46135 46836 47014 48333 49338 49379 49413 49592 51658 53413 53804 57318 60957 61108 62095 62361 62633 63493 66201 67931 69189 72417 72523 72761 73904 85856 92070 92226 93158 93485 93968 95817 96199 98580 98783 |
| Other cardiac procedures | K23.4 K38.1 K38.2 K45.2 K45.3 K45.4 K45.5 K46 K46.1 K46.2 K46.3 K46.4 K46.8 K46.9 K47 K47.1 K47.2 K47.3 K47.4 K47.5 K47.8 K47.9 K48 K48.1 K48.2 K48.3 K48.4 K48.8 K48.9 K53 K53.1 K53.2 K53.8 K53.9 K55.3 K55.4 K55.8 K55.9 K66 K66.8 K66.9 | 2818 3140 3827 5904 6557 10603 15769 18903 19164 22020 22733 30993 31512 31571 33620 34904 38384 39810 41757 43492 44723 47788 48206 48207 48822 51702 53652 53709 54275 61592 64723 69247 70609 92233 93182 93432 94783 95382 |

Table S2: Clinical codes used for defining cardiac surgery, AF and comorbidity in the study.

| **Exposure and covariates** | **Definitions** |
| --- | --- |
| AF | <https://www.caliberresearch.org/portal/show/af_hes>  <https://www.caliberresearch.org/portal/show/af_gprd> (categories 4,5,6) |
| Smoking status | https://caliberresearch.org/portal/show/smoking_status_gprd<https://caliberresearch.org/portal/show/smoking_status_gprd> (categories 2,3,4)  ICD10: F17 |
| Diabetes | <https://www.caliberresearch.org/portal/show/dm_gprd> (categories 3,4,6)  <https://www.caliberresearch.org/portal/show/dm_hes> (categories 3,4,6) |
| Hypertension | <https://www.caliberresearch.org/portal/show/ht_gprd> (categories 3,4)  <https://www.caliberresearch.org/portal/show/ht_hes> (categories 3,4) |
| Stable angina | <https://www.caliberresearch.org/portal/show/sa_diagnosis_gprd> (category 4)  <https://www.caliberresearch.org/portal/show/angina_hes>https://www.caliberresearch.org/portal/show/angina_hes |
| Unstable angina | <https://www.caliberresearch.org/portal/show/unangina_gprd> (category 3)  ICD10: I20.0, I24.0, I24.8, I24.9 |
| Myocardial infarction | https://www.caliberresearch.org/portal/show/myo_infarct_gprd<https://www.caliberresearch.org/portal/show/myo_infarct_gprd> (categories 3,4,5)  ICD10: I21 |
| stroke | <https://www.caliberresearch.org/portal/show/ischaemic_stroke_gprd>(category 3)  <https://www.caliberresearch.org/portal/show/haem_stroke_gprd> (categories 3-8)  <https://www.caliberresearch.org/portal/show/stroke_nos_gprd> (category 3)  ICD10: I60, I61, I63, I64, I62.0, I62.1, I62.9, G46.3, G46.4, G46.5, G46.6, G46.7, |
| Dementia | https://www.caliberresearch.org/portal/show/dementia_gprd<https://www.caliberresearch.org/portal/show/dementia_hes> (categories 2-5)  https://www.caliberresearch.org/portal/show/dementia_hes<https://www.caliberresearch.org/portal/show/dementia_gprd> (categories 2-5) |
| heart failure | <https://www.caliberresearch.org/portal/show/hf_gprd> (categories 3,4,5,6)  https://www.caliberresearch.org/portal/show/hf_hes |
| Chronic obstructive pulmonary disease | https://www.caliberresearch.org/portal/show/copd_gprd<https://www.caliberresearch.org/portal/show/copd_gprd> (categories 3,5) <https://www.caliberresearch.org/portal/show/copd_hes> (categories 3,5) |
| chronic kidney disease | [https://www.caliberresearch.org/portal/show/renal_gprd (categories 3-7) https://www.caliberresearch.org/portal/show/renal_hes](https://www.caliberresearch.org/portal/show/renal_hes) (categories 3-7) |
| cancer | https://www.caliberresearch.org/portal/show/cancer_gprd  https://www.caliberresearch.org/portal/show/cancer_hes |
| Asthma | ICD10 J45, J46 and corresponding Read code |
| Valvular disease | ICD10: I05. I06. I07, I08, I34, I35, I36, I37 and corresponding Read code. |
| Deep vein thrombosis | <https://www.caliberresearch.org/portal/show/dvt_gprd>  https://www.caliberresearch.org/portal/show/dvt_hes |
| Pulmonary embolism | <https://www.caliberresearch.org/portal/show/pe_gprd>  https://www.caliberresearch.org/portal/show/pe_hes |
| Mitral valve disease | ICD10 I05, I34 and corresponding Read code |
| Supraventricular tachycardia | ICD10 I47.1 and corresponding Read code |
| Cardiogenic shock | ICD10 R57 and corresponding Read code |
| Ventricular tachycardia | ICD10 I47.9, I47.2, R00.0 and corresponding Read code |
| Transient ischemic attack | <https://www.caliberresearch.org/portal/show/tia_gprd>  <https://www.caliberresearch.org/portal/show/tia_hes> |
| Hypothyroidism | <https://www.caliberresearch.org/portal/show/hypothyroid_gprd>  https://www.caliberresearch.org/portal/show/hypothyroid_hes |
| Dyslipidaemia | <https://www.caliberresearch.org/portal/show/dyslipid_gprd>  https://www.caliberresearch.org/portal/show/dyslipid_hes |
| Anticoagulants | https://www.caliberresearch.org/portal/show/anticoagulants_and_protamine_gprdprod |
| Antiplatelet drugs | https://www.caliberresearch.org/portal/show/antiplatelet_drugs_gprdprod |
| Anti-arrhythmic drugs | https://www.caliberresearch.org/portal/show/anti_arrhythmic_drugs_gprdprod |
| Beta adrenoceptor blocking drugs | https://www.caliberresearch.org/portal/show/beta_adrenoceptor_blocking_drugs_gprdprod |
| Diuretics | https://www.caliberresearch.org/portal/show/diuretics_gprdprod |
| Warfarin | https://www.caliberresearch.org/portal/show/hcm_warfa_gprdprod |
| Hypertension and heart failure drugs | https://www.caliberresearch.org/portal/show/hypertension_and_heart_failure_gprdprod |
| Statin | https://www.caliberresearch.org/portal/show/lipid_regulating_drugs_gprdprod |
| NSAIDS | https://www.caliberresearch.org/portal/show/nsaids_gprdprod |
| Cardiac glycosides | Complete medication list available upon request. |
| Immunosuppressants |  |
| Inotropic drugs |  |
|  |  |

Table S3: Distribution of patients through the different predicted AFACS risk categories according to valvular heart disease status at baseline

| Predicted risk of AFACS  (quartiles) | Valvular heart disease at baseline, n (%) | No Valvular heart disease at baseline, n (%) |
| --- | --- | --- |
| Lowest | 773 (10.0) | 7657 (29.8) |
| Low | 1034 (13.3) | 7242 (28.2) |
| High | 1682 (21.7) | 6724 (26.2) |
| Highest | 4261 (55.0) | 4091 (15.9) |
|  | Total: 7750 (100) | Total: 25714 (100) |

Table S4: Distribution of confirmed AFACS cases by predicted risk groups in patients with baseline valvular heart disease

| Predicted risk of AFACS  (quartiles) | Detected AFACS cases, n (%), in patients with valvular heart disease at baseline |
| --- | --- |
| Lowest | 88 (4.0) |
| Low | 224 (10.1) |
| High | 422 (19.1) |
| Highest | 1480 (66.9) |
|  | Total: 2214 (100) |

Table S5: Subsequent stroke occurrences in the different predicted AFACS risk groups

| Predicted risk of AFACS (quartiles) | Stroke occurrence during follow-up, n (%) | % of Stroke during follow-up per risk quartile |
| --- | --- | --- |
| Lowest | 292 (10.5) | 3.46 |
| Low | 487 (17.5) | 5.88 |
| High | 833 (30.0) | 9.91 |
| Highest | 1165 (42.0) | 13.95 |
|  | Total: 2514 (100) |  |

Figure S1: Importance of each predictor assessed by mean decrease accuracy and mean decrease Gini.


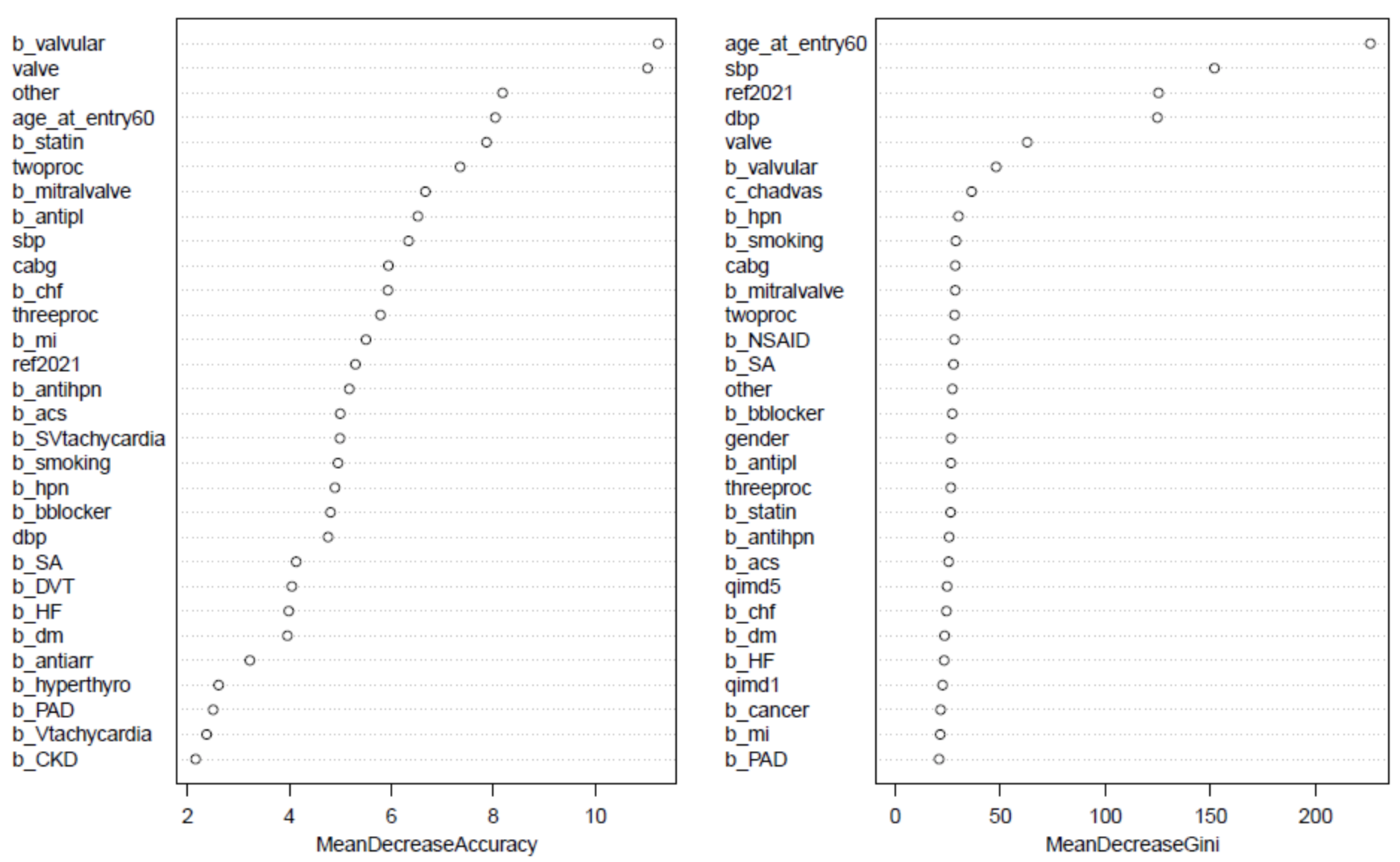


Figure S2: The ROC (Receiver Operating Characteristic) curve for the random forest classifier performance.


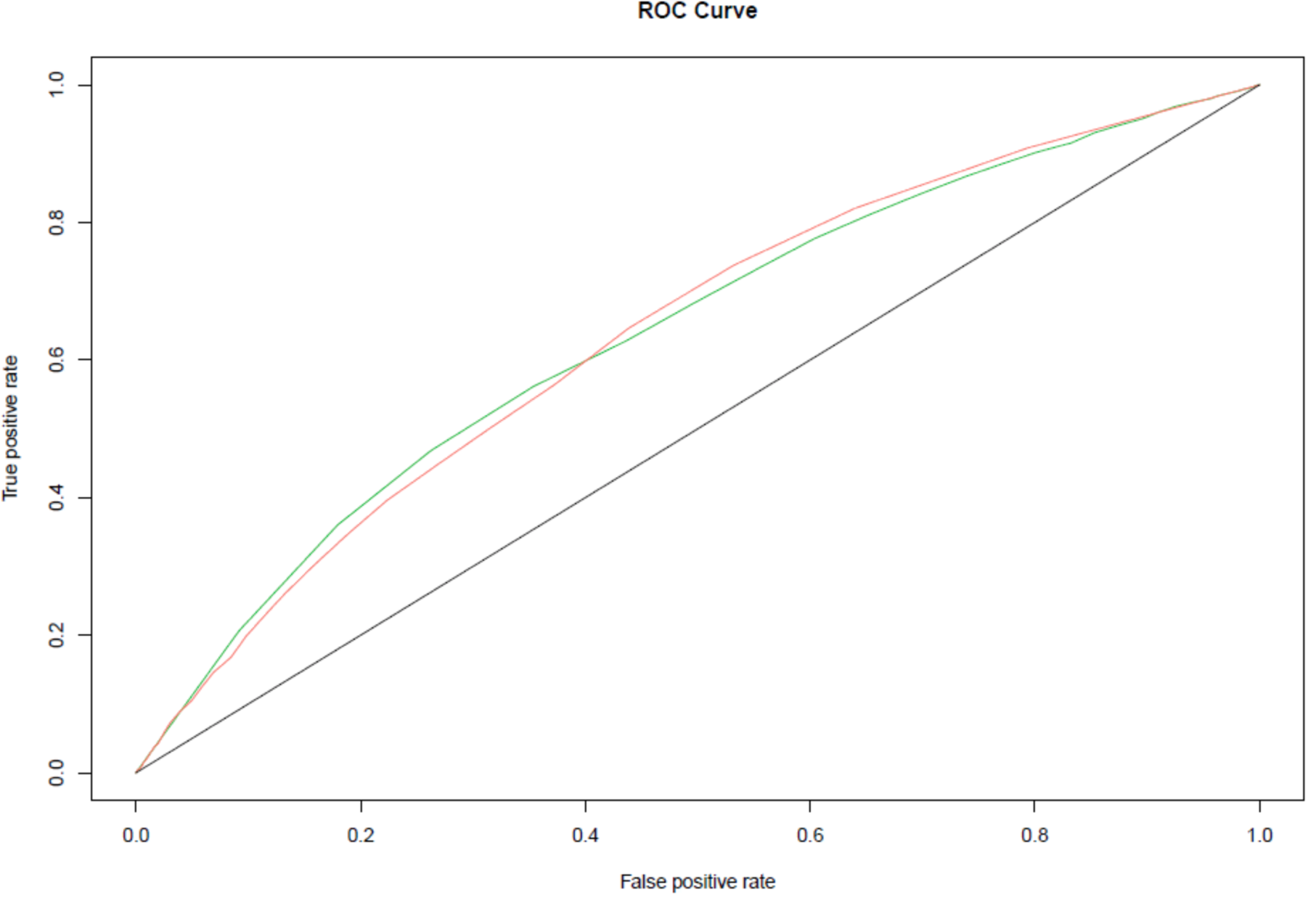


*Red line (incident atrial fibrillation as the outcome) Green line (free from atrial fibrillation as the outcome).
